# Supplementary material for: Comparative Whole Genome Analysis of an Anaplasma phagocytophilum Strain Isolated from Norwegian Sheep
Source: Pathogens. 2022 May 21;11(5):601. doi: 10.3390/pathogens11050601 (PMC9146208; doi:10.3390/pathogens11050601)
Supplement: Supplementary file 1 [file pathogens-11-00601-s001.zip › Supplementary_Figure_S1.pdf]

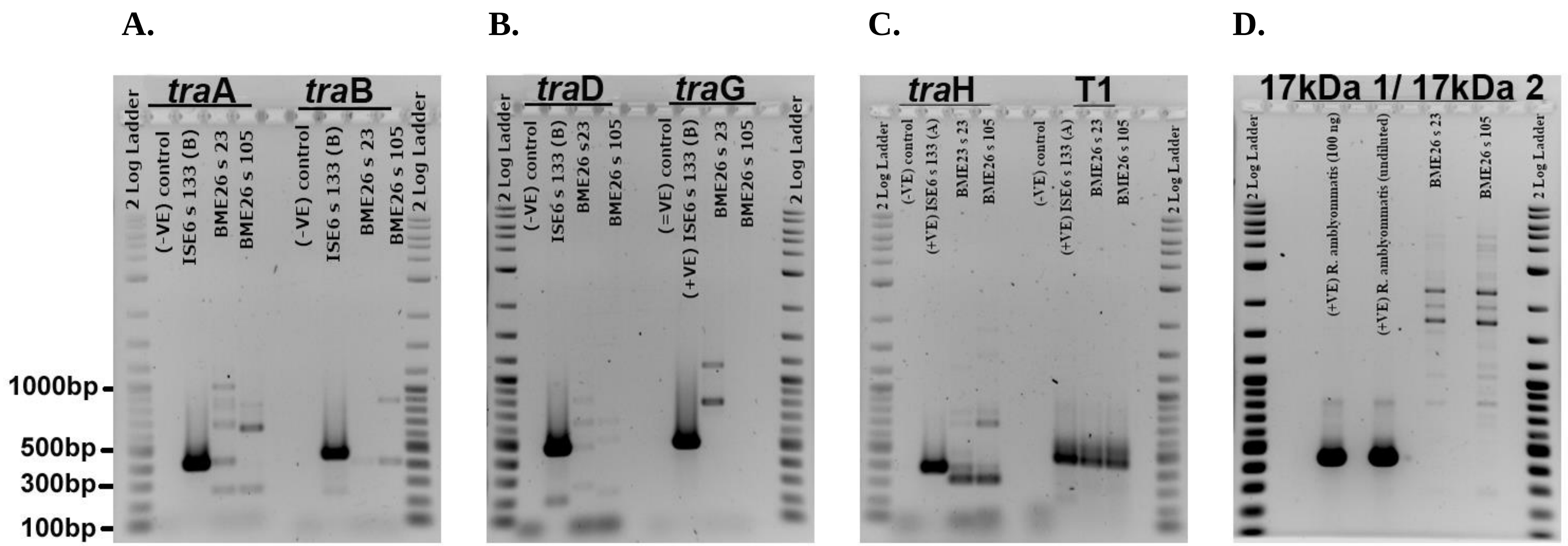

Supplementary Figure S1. Agarose gel electrophoresis of PCR analysis targeting *Rickettsia tra* genes in different tick cell lines. Representative agarose gel analysis showing that *Rickettsia-traA*, *traB*, *traD*, *traG* and *traH* genes were only found in the ISE6 tick cell line, panels **A**, **B** and **C**. Initially, we detected *traA* and *traD* amplicons in the *Rhipicephalus microplus* tick cell line BME26 passage number 23. However, these results appear to indicate contamination with DNA extracted from ISE6 as similar size amplicons were not detected in a later passage (s 105). The *Aedes vexans* mosquito-embryo cells were used as negative (-VE) and inhibition control (+ VE) for Rickettsia *tra* genes, tick β-actine genes (T1), and the *Rickettsia*-genus specific *17kDa* antigen gene.
