# Supplementary figures and images for: Comparative Whole Genome Analysis of an Anaplasma phagocytophilum Strain Isolated from Norwegian Sheep
Source: Pathogens. 2022 May 21;11(5):601. doi: 10.3390/pathogens11050601 (PMC9146208; doi:10.3390/pathogens11050601)

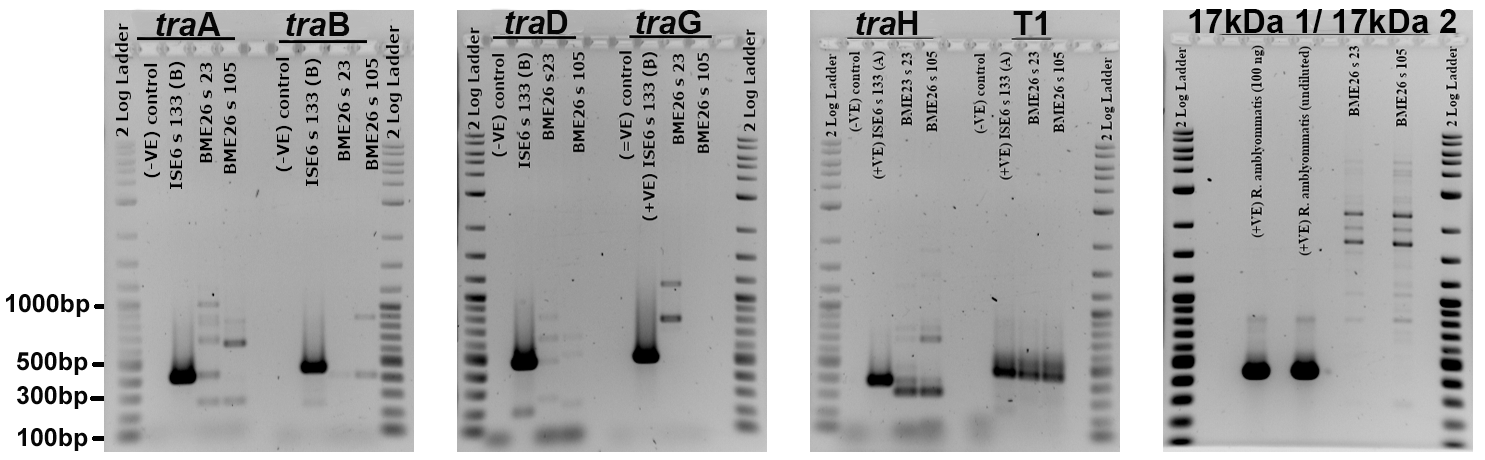

Supplement: Supplementary file 1 [file pathogens-11-00601-s001.zip › Supplementary Figure S1.tif]
